# Infectious or Recovered? Optimizing the Infectious Disease Detection Process for Epidemic Control and Prevention Based on Social Media

**DOI:** 10.3390/ijerph17186853

**Published:** 2020-09-19

**Authors:** Siqing Shan, Qi Yan, Yigang Wei

**Affiliations:** 1 School of Economics and Management, Beihang University, Beijing 100191, China; shansiqing@buaa.edu.cn (S.S.); weiyg@buaa.edu.cn (Y.W.); 2 Beijing Key Laboratory of Emergency Support Simulation Technologies for City Operation, Beijing 100191, China

**Keywords:** social media, flu, disease detection, sentiment analysis, text classification

## Abstract

Detecting the period of a disease is of great importance to building information management capacity in disease control and prevention. This paper aims to optimize the disease surveillance process by further identifying the infectious or recovered period of flu cases through social media. Specifically, this paper explores the potential of using public sentiment to detect flu periods at word level. At text level, we constructed a deep learning method to classify the flu period and improve the classification result with sentiment polarity. Three important findings are revealed. Firstly, bloggers in different periods express significantly different sentiments. Blogger sentiments in the recovered period are more positive than in the infectious period when measured by the interclass distance. Secondly, the optimized disease detection process can substantially improve the classification accuracy of flu periods from 0.876 to 0.926. Thirdly, our experimental results confirm that sentiment classification plays a crucial role in accuracy improvement. Precise identification of disease periods enhances the channels for the disease surveillance processes. Therefore, a disease outbreak can be predicted credibly when a larger population is monitored. The research method proposed in our work also provides decision making reference for proactive and effective epidemic control and prevention in real time.

## 1. Introduction

**Figure 1:**
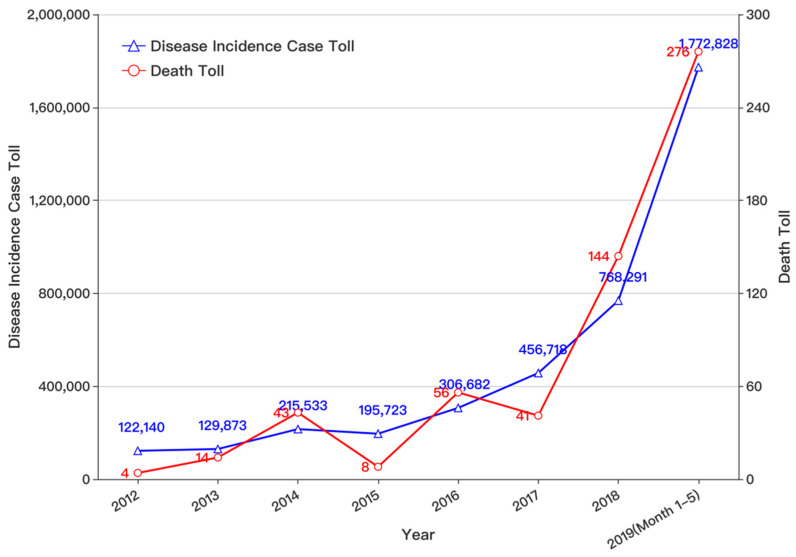
Number of People in the Incidences and Deaths from Flu in China.

The traditional infectious disease detection process is being challenged by potential social media applications [1,2]. The latest estimates released by the United States Centers for Disease Control and Prevention (US-CDC) revealed the worldwide severity of the illness. According to this authoritative report, the US-CDC estimates that in the period between 1 October 2018 and 4 May 2019, there were approximately 37.4 million to 42.9 million flu infectious in the population population, among which there were from 17.3 to 20.1 million flu-related medical visits [3]. Furthermore, 531,000 to 647,000 people require flu-related hospitalizations, and unfortunately, influenza caused 36,400–61,200 estimated deaths. This estimate is based on more recent data from a larger and more diverse group of countries, including lower middle-income countries, and this estimate excludes deaths from non-respiratory diseases. The statistics indicate a severe reality and a pressing challenge because influenza causes significant losses of human life and damage to property worldwide. As evidenced by the current flu season, influenza viruses can rapidly mutate, evading the most current vaccine formulations [4]. Infectious diseases continue to be the leading cause of death worldwide, and they cause serious loss of life and property when they cannot be quickly and accurately assessed [5]. The World Health Organization (WHO) announced on 15 August 2003 that by 7 August 2003, there were 8422 infectious cases of Severe Acute Respiratory Syndrome (SARS) worldwide, which involved 32 countries and regions [6]. The number of deaths due to SARS in the world totaled 919, and the mortality rate was almost 11%. A handful of studies estimated the global macroeconomic impact of SARS at USD 30–100 billion, or approximately USD 3–10 million per case [7]. However, from the first reported case on 15 December 2002 to the first epidemic announcement by the Chinese government on 11 February 2003, the time span was almost two months. The consequence was a significant loss of life and property due to the lack of effective disease identification and monitoring methods for real-time epidemic control. Based on the infectious disease epidemic report issued by the National Health Commission of China, in recent years, the number of incidences and deaths due to influenza in C-class infectious diseases shows an explosively upward trend annually (see Figure 1). The death toll for five months during 2019 was nearly two times that for all of 2018.

The fundamental cause of these serious outbreaks is summarized as follows. First, traditional disease prevention and control institutions mainly rely on a single channel for information monitoring and access. Specifically, data are exclusively sourced from clinical statistics. However, relying exclusively on clinical statistics has obvious disadvantages, such as being time consuming and creating high labor costs [8,9]. The traditional detection methods cannot integrate multichannel infectious disease information, such as social media and search engine data. Second, social media data are a powerful and promising tool that have been applied to many research subjects, such as healthcare informatics [10,11], sentiment analytics [12], and disaster management [13,14,15]. The remarkable value of social media has been widely recognized [16]. Particularly in this paper, we refer to microblogs posted on social media platforms especially from Sina Weibo as weibos. Although disease prevention and control institutions have exerted a significant role in disease detection, they might come up against substantial difficulties in using social media data in disease detection and control due to the lack of analytic methods or accurate monitoring of outbreaks and periods of infectious diseases. There is still a long journey to go to fully utilize social media data in disease prevention and control. Third, the flu is characterized by strong contagiousness and rapid spread, which makes it difficult to monitor in real-time or precisely estimate the spread of the flu. For example, the flu can be easily spread by droplets or contaminated items in the air and contact among people. The Centers for Disease Control and Prevention (CDC) publishes data on influenza-like illness ILI based on statistics and evaluation after a patient’s visit. It would be extremely difficult to obtain information and perform an analysis before the visit. However, apparently, time lags could lead to delayed treatment. Fourth, Google Flu Trends (GFT) provided an estimate of more than double the proportion of clinical data for influenza-like illness (ILI) published by the Centers for Disease Control and Prevention (CDC) [17]. The ILI was calculated based on surveillance reports from laboratories across the United States [18]. Google used web search data to propose a GFT model for real-time monitoring. When patients are aware of their active flu period and search for flu-related keywords through search engines such as Google, the patients’ behavior is recorded by the search engine. Social media sensors, in contrast, show unique advantages for quick flu monitoring and reliable estimation and prediction. There has been a growing consensus that social media sensors can also perform real-time monitoring that is more accurate than GFT [8]. Fifth, part of the ILI data cannot be collected and thus are not available if the patient does not go to the hospital, which makes disease monitoring information inaccurate and incomplete. Therefore, the severity and urgency of the disease as reflected by traditional statistics are often underestimated. However, social media can capture this part of the data because when bloggers catch a cold, they can post their own flu symptoms through social media. If bloggers do not realize that they catch flu, they can also post some flu-related microblogs on social media. This part of the data can also be seen in advance of CDC released reports [19]. Sixth, the previous literature mainly focused on whether the bloggers are infected based on the social media platform [20] and cannot accurately distinguish the various periods of the disease, which largely reduces the effectiveness and pertinence of the information for disease control measures.

This section provides an extensive review of the relevant literature in three parts: optimization of the infectious disease detecting process, social media utilization for disease detection and semantic analysis techniques based on social media data. Much of the current research focuses on social media to analyze short texts [21,22,23], in which several papers predict flu trends by classifying flu-related social media data [19]. However, these studies do not divide the flu periods any further. Speedily evolving infectious diseases, including SARS, Ebola and influenza, pose significant health threats throughout the world because of their rapidly changing status and complicated detection process [24,25]. A considerable amount of work is devoted to forecasting disease outbreaks. A two-period model optimized the process of when and where to assign Ebola treatment units across geographic regions during the outbreak’s early phases [26]. Chen et al. (2018) developed a mixed-integer programming (MIP)-based framework to systematically analyze a rich set of policies and to determine the optimal hepatocellular carcinoma surveillance policies that maximize the societal net benefit [27]. It is observed that the surveillance policies should be adapted to different disease progression rates and states. Several studies in this area focused on finding optimal surveillance solutions for flu vaccine production and allocation [28,29]. Another study considered the conditions of limited reporting and spatial aggregation on the optimization of influenza surveillance system design [30]. Research in this area is mainly concerned with epidemiological inference and prediction based on clinical data collection, but few of the studies provide improved detecting measures using social media data, which represent an alternative source of passive traditional surveillance data that have a larger volume and fewer reporting delays.

Data from social networks show apparent advantages in several aspects, such as being real time and time-sharing, along with a broad scope of data coverage [31,32,33,34]. Disease surveillance has been investigated based on the recent rise in the popularity and scale of social media data. Aiello et al. (2011) reviewed and addressed the use, promise, perils, and ethics of social media- and internet-based data collection for public health surveillance [35]. Additionally, the infectious disease detection process is being challenged by the potential applications of social media [1,2,36]. Multiple types of social media have become emerging and promising data sources of disease surveillance and shown advanced achievements in tracking health informatics in different areas all over the world. Raamkumar et al. (2020) examined the differences of COVID-19-related public responses on Facebook in the United States, England and Singapore and showed that social media analysis was capable of providing insights about the communication strategies during disease outbreaks [37]. Lwin et al. (2018) and Vijaykumar et al. (2017) investigates how Facebook can be utilized to implement and adapt in responding to the Zika epidemic in Singapore [38,39]. Moreover, Dubey et al. (2014) identified and evaluated YouTube as significant resource for providing and disseminating information on public health issues like West Nile virus infection [40]. Davidson et al. (2015) constructed an empirical network to substantially improve performance in predicting infections one week into the future using CDC data and combining this with internet-based data in the U.S. [41]. Chen et al. (2016) proposed two temporal topic models to capture hidden states of a flu-related user and get better flu-peak predictions by using Twitter data. In addition, they validated their approaches by modeling the flu using Twitter in multiple countries of South America [42]. Lamb et al. (2013) demonstrated that the use of Twitter data leads to significant improvements in flu surveillance by discriminating those categories of flu tweets that reported infection from those that expressed concerned awareness of the flu as well as tweets about the authors versus those about others [43].

Sentiment classification [44,45], feature extraction [46] and public opinion monitoring [47,48] are performed based on a social network dataset for sentiment analysis. Chen et al. (2020) introduced a novel approach of adding semantics as additional features into the training set for sentiment analysis, and they applied this approach to predict sentiment for three different Twitter datasets [49]. The authors also investigated the real-time flu detection problems and proposed a flu detection model with emotional factors and semantic information [50]. Adamopoulos et al. (2018) examined the effect of latent personality traits on consumers’ behavior and preferences, which originated from social media users’ levels of emotional range [51]. The effect of social media advertising content on customer engagement was also studied via Facebook users’ humor and emotion [52]. Although sentiment analysis has been widely applied to many fields, few researchers consider it to be a powerful tool to be used in determining a patient’s status when detecting infectious diseases. Furthermore, word embedding techniques, such as bag of words [53] and word2vec [54], have become effective means of text processing. High-quality word vector representations provide distributional information about words [55], especially for word2vector, which appears to be outstanding at improving a model’s performance on a limited amount of data. The development of word2vector significantly improves the effectiveness of word representations by transforming sparse, discrete and high-dimensional vectors into dense, continuous and low-dimensional vectors [56]. It is a foundation to transform word segments into fixed dimensional vectors, namely, word embedding, when studying user-generated content [57,58]. In this paper, the use of high dimensional numeral vectors to represent words serve as semantic feature extractors or the input variables of a neural network. Artificial Neural Network (ANN) techniques have been used widely in text classification. Hughes et al. (2017) have used Convolutional Neural Networks (CNNs) for text processing and classification in online news, reviews and medical text [59]. A large number of modified ANN techniques have emerged with technical advancements. Recurrent Neural Networks (RNNs) have also been an effective method for speech recognition and text sequence tagging [60], and Long Short Term Memory (LSTM) networks [61] perform well for sequence-based learning tasks. In addition, a large amount of text processing research has emerged, including the use of part-of-speech tagging [62], lexicon approaches [63], and other deep learning techniques [64]. The extant literature has several key limitations. Firstly, the abovementioned studies have mainly focused on a single aspect of text processing, sentiment classification or flu tracking [43,62]. More importantly, these papers do not adequately consider the influence of sentiment polarity on the classification of flu-related weibos or on dividing the different periods of flu-related weibos [50]. Therefore, this paper incorporates sentiment factors into flu surveillance research, and the period classifications of flu-related weibos are probed. Second, neural network techniques are used to process the weibos at the text level [61,65]. LSTM networks can be used for sentiment analysis of film reviews, part-of-speech tagging, and other fields [60]. Previous studies show that LSTM performs relatively well in text processing but has been rarely used for disease weibo analysis. To fill in this gap, this paper aims to investigate the relationship between sentiment polarity and the flu period at the word level and text level based on a weibo dataset.

The main research rationale of this study is straightforward, i.e., first, to investigate the relationship between the sentiment polarity and the flu period from social networks, and second, to optimize the disease detecting process by predicting the different periods of flu.

## 2. Materials and Methods

### 2.1. Research Model

**Figure 2:**
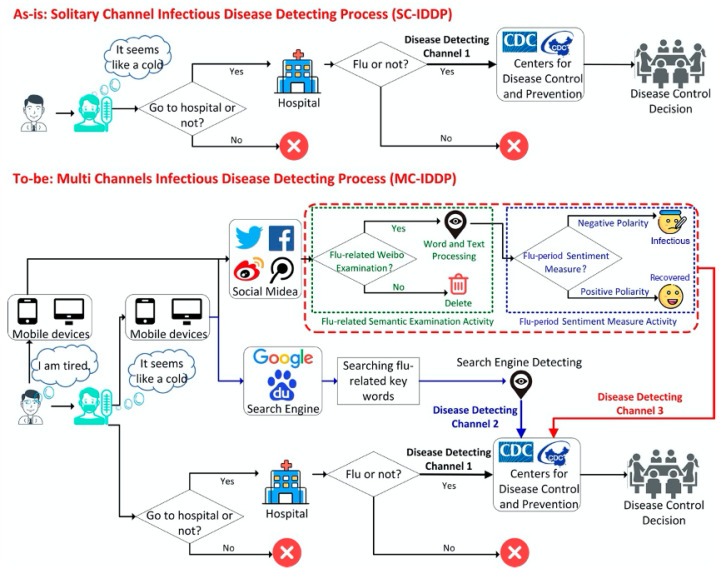
Solitary Channel and Multi-Channels Process.

The model proposed in this paper can detect infectious diseases through multiple channels; they can be perceived earlier and the model larger populations and more efficiency than traditional processes (See Figure 2).

The upper part of Figure 2 is a schematic diagram of a traditional solitary infectious disease detecting process. Patients usually go to the hospital when they have already suffered influenza. Only afterwards it is possible for the CDC to detect the flu trends of the whole society. In the “as-is” process, there is only one channel to detect infectious diseases, which is through hospital institutions. The “as-is” process is abbreviated as SC-IDDP. The “to-be” process is the multiple channel infectious disease detection process (MC-IDDP). The bottom part of Figure 2 illustrates the MC-IDDP process based on social media platforms. MC-IDDP has three infectious disease detecting channels. Channel 1 is the traditional infectious disease detecting channel, mentioned above. Channel 2 monitors the infectious diseases by using search engines [17,18]. This paper constructs Channel 3 to detect infectious diseases using social media data. Compared with Channel 1 and 2, Channel 3 enables more effective disease detection for the following three reasons. First, the traditional Channel 1 is not able to use social media data to detect diseases, such as flu, whereas Channel 2 and Channel 3 can use these data. Second, search engines can detect the keywords of the corresponding flu symptoms and treatments in Channel 2 when people realize that they have the flu. It neglects some of the patients who have caught the flu but do not search for flu keywords and simultaneously adds fake patients who run the search engine for flu keywords without flu. Third, although people do not realize that they have caught the flu, they usually post tweets via social media to reflect their feelings, opinions and behaviors, which can be detected by Channel 3. It helps the CDC to analyze social media data to discover early flu trends. Additionally, Channel 3 can not only monitor infectious disease outbreaks in early stages but also identify the flu period. The content inside the dotted line (shown in Figure 2) is the main research content of this paper, which aims to find the flu-related weibos and further determine the flu period to improve the accuracy of infectious disease detection.

As the “to-be” process optimized by social media, the MC-IDDP process can improve the reliability of detecting infectious disease and discover more infectious people. It recognizes the disease periods earlier and works in an efficient way. Next, we propose 4 propositions and corresponding demonstrations.

**Proposition** **1.** *The MC-IDDP process detects a larger number of potentially infectious people in the population.*

**Figure 3:**
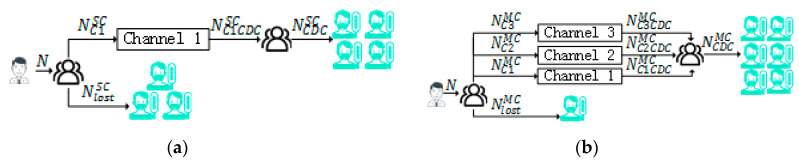
The Logical Structure Model of a Solitary Channel and Multiple Channels. (**a**) Solitary Channel; (**b**) Multiple Channels.

**Demonstration** **1.** *The logical structure model of MC-IDDP is shown in Figure 3. Figure 3a shows the logical structure model of a solitary channel, and Figure 3b shows the logical structure model for multiple channels.*

The main parameters are described as follows. N represents the number of possible infectious people; $N_{C1}^{SC}$ represents the number of possibly infectious people recorded by hospitals using a solitary channel; $N_{lost}^{SC}$ represents the number of possibly infectious people but do not go to the hospital in a solitary channel; $N_{C1CDC}^{SC}$ represents the number of infectious people who are diagnosed and reported to the CDC in a solitary channel; $N_{C1}^{MC}$ represents the number of possibly infectious people recorded by hospital in multiple channels; $N_{C1CDC}^{MC}$ represents the number of infectious people who are diagnosed and reported to the CDC in Channel 1; $N_{C2}^{MC}$ represents the number of possible patients who search disease-related information through search engines in a multiple channel environment; $N_{C2CDC}^{MC}$ represents the number of infectious people who are detected and reported to the CDC after analyzing the search data in Channel 2; $N_{C3}^{MC}$ represents the number of possible patients who post related microblogs through social media in a multiple channel environment; $N_{C3CDC}^{MC}$ represents the number of infectious people who are detected and reported to the CDC after analyzing social media data in Channel 3; $N_{lost}^{MC}$ represents the number of possible patients who do no go to the hospital, and do not use search engines and social media in multiple channels; $N_{CDC}^{SC}$ represents the number of infectious people who are diagnosed and reported to the CDC in a solitary channel; $N_{CDC}^{MC}$ represents the number of infectious people who are detected by the three channels and reported to the CDC.

Obviously, in a solitary channel,

(1) $$N=N_{C1}^{SC}+N_{lost}^{SC}$$

In multiple channels,

(2) $$N=N_{C1}^{MC}\cup N_{C2}^{MC}\cup N_{C3}^{MC}+N_{lost}^{MC}$$

Without the loss of generality, it is assumed that the number of infectious people who are admitted to the hospital remains the same regardless of whether it is a solitary channel or multiple channels, which is,

(3) $$N_{C1}^{MC}=N_{C1}^{SC}$$

The following relationship exists,

(4) $$N_{C1}^{MC}\cup N_{C2}^{MC}\cup N_{C3}^{MC}>N_{C1}^{SC}$$

According to Formula (4), in multiple channels, more infectious people can be detected. Proposition 1 is validated.

**Proposition** **2.** *The MC-IDDP process recognizes the patients early and detects the infectious disease in time.*

**Figure 4:**
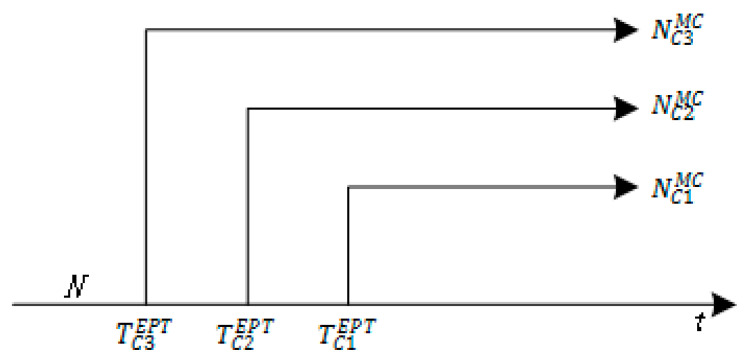
The Logical Structure Model over Time.

**Demonstration** **2.** *The Google flu trends model provides a promising measure in that the search engine records the users’ relevant search data on disease symptoms and treatments, to detect infectious diseases. Additionally, some of the patients do not realize that they might be infected but post weibos with disease symptoms. Therefore, people’s sentiment and physical conditions can be reflected in social media, which is an important infectious disease sensor. We added time information in Figure 3b to generate Figure 4. The horizontal axis t indicates the earliest time at which each channel can detect infectious disease information.*

In Figure 4, EPT represents the Earliest Possible Time, and $T_{Ci}^{EPT}$ represents the earliest possible time to detect the disease by Channel i. Obviously, we conclude that

(5) $$T_{C3}^{EPT}<T_{C2}^{EPT}<T_{C1}^{EPT}$$

According to Formula (5), the EPT for Channel 3 is the smallest, and the EPT for Channel 1 is the largest. Therefore, the MC-IDDP process based on social media data can achieve more timely monitoring.

**Proposition** **3.** *The MC-IDDP process can conduct infectious disease detection more efficiently.*

**Demonstration** **3.** *In the MC-IDDP process, Channel 1 requires a large number of doctors and staff with high operating costs, but Channel 2 and Channel 3 rely only on big data and analytical tools to conduct the surveillance. Compared with Channel 1, the operation costs of Channel 2 and Channel 3 can be negligible. Therefore, the total cost of the MC-IDDP process is almost the same as the total cost of the SC-IDDP process. However, the MC-IDDP process can achieve a wider range of detection (according to Proposition 1), and thus, the MC-IDDP process has higher monitoring efficiency. Proposition 3 is validated. The Detection Accuracy is defined as the percentage of the correct number of patients out of the total number of possible patients who have been monitored through the infectious disease detection channels. In this paper, the Detection Accuracy ($Acc_{D}$) can be calculated by the following equation,*

(6) $$Acc_{D}=\frac{TP+TN}{TP+TN+FP+FN}$$

*where TP is an abbreviation of True Positive, which indicates that an infectious patient is diagnosed as a patient; TN is an abbreviation of True Negative, which means that a nonpatient is diagnosed as a nonpatient; FP is an abbreviation of False Positive, which indicates that a nonpatient is misdiagnosed as a patient (Type I error); FN is an abbreviation for False Negative, which means that a real patient is misdiagnosed as a nonpatient (Type II error).*

**Proposition** **4.** *The detection accuracy in any channel of the MC-IDDP process helps to improve the entire accuracy of the disease detection.*

**Demonstration** **4.** *The MC-IDDP process has three disease detection channels. Each channel has independent methods and techniques. The detection accuracy of Channel 1 depends on the hospital’s medical plan and medical technology. The detection accuracy of Channel 2 depends on the statistical analysis and technical means used by the search engine. The detection accuracy of Channel 3 depends on the semantic analysis and machine learning application to social media data. The detection results of the three channels do not affect one another, and thus, the detection accuracy of each channel is related to only the method and supporting technology of that channel. Increasing the detection accuracy in any channel can improve the entire accuracy of the disease detection. The proposition is validated.*

This paper focuses on detection Channel 3. To improve the detection accuracy of Channel 3 using social media data, this paper proposes an effective dual analytical activity model to determine the status and period of the infected population. The next section discusses the infectious disease detection Channel 3 and the main activities.

### 2.2. Infectious Disease Detection Channel 3

**Figure 5:**
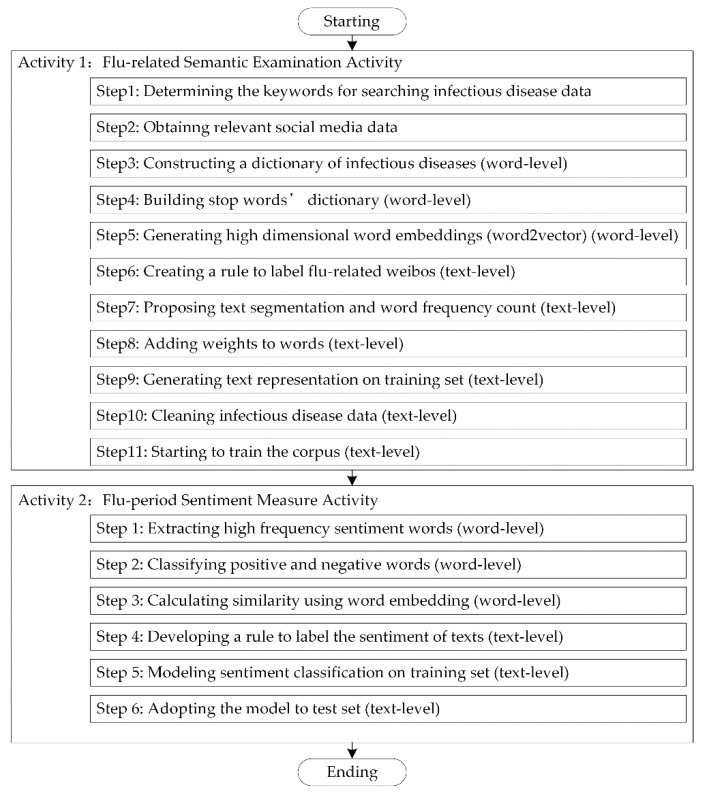
Dual Analytical Activity Model in Channel 3.

The Infectious Diseases Detection Channel 3 is a crucial channel for the MC-IDDP process that makes direct use of social media data. It has two main activities: flu-related semantic examination activity and flu-period sentiment measure activity, and thus, Channel 3 is also named the dual analytical activity model, as shown in Figure 5. The contents and features of the model are described in detail in the following section.

#### 2.2.1. Flu-Related Semantic Examination Activity

The purpose of the Flu-related semantic examination activity is to enable semantic recognition and analysis of social media data based on infectious diseases (taking the flu as an example). The main content of the activity includes at least 12 steps, such as determining keywords for searching infectious disease data and obtaining relevant social media data, as shown in Figure 5. Next, we provide some crucial steps.

1. Obtaining Flu Data Based on Social Media

Six keywords were crawled from Sina Weibo; these six keywords include “flu (Gan Mao)”, “influenza (Liu Gan)”, “cough (Ke Sou)”, “fever (Fa Shao)”, “sneeze (Pen Ti)” and “nasal congestion (Bi Sai)”. We used Python for this task, and these flu-related weibos constitute an elementary corpus.

2. Cleaning the Flu Data

**Figure 6:**
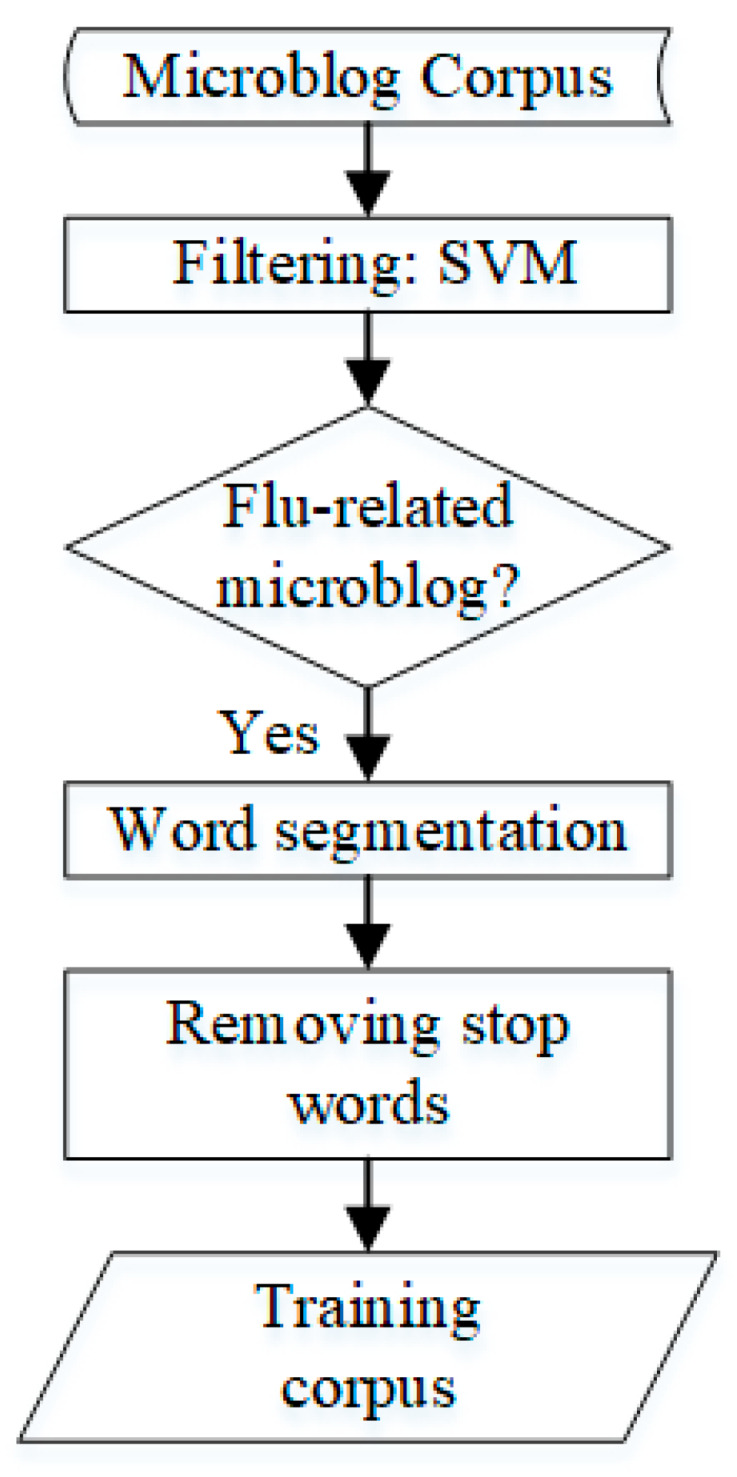
Cleaning Data.

Considering the existence of advertisements and forwarding, this study used Support Vector Machine (SVM) to screen the invalid data and retain the flu-related weibos. The final flu-related weibo corpus is generated after word segmentation and the removal of stop words. The entire process is shown in Figure 6.

#### 2.2.2. Flu-Period Sentiment Measure Activity

This activity is mainly to accurately identify the patient’s disease period to improve the detection accuracy of infectious diseases. The whole process includes six steps. Activity 2 in Figure 5 shows that the word level detection consists of 3 steps and the text level includes 3 steps, which is introduced as follows.

1. Relationships between Sentiment Polarity and Flu Period at the Word Level

**Figure 7:**
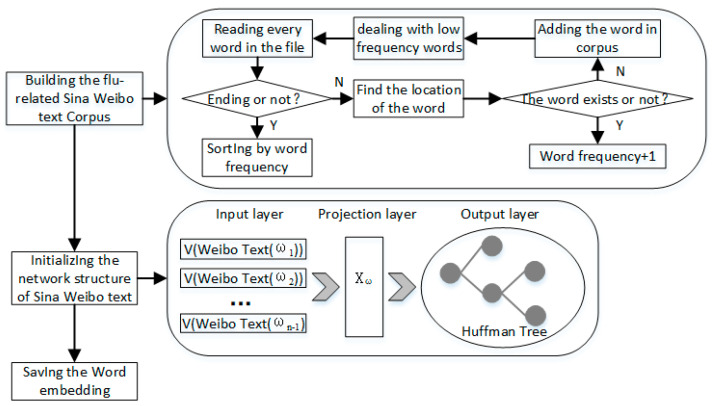
Word2vector Principles of Sina Weibo Text.

Word2vector is an efficient training method to transform a symbol into a structure and digital representation. Word embedding is represented differently in different vocabularies or by different training methods. The principles of Word2vector are mainly separated into three parts to transform the words from the vocabulary of a flu-related weibo into high-dimensional space vectors, as follows in Figure 7.

- Building the vocabulary of the flu-related weibo texts: the processing of the text, which means that a specific vocabulary is required;
- Initializing the network structure of the weibo text: the initialization of parameters in the CBOW model, with Huffman coding generation;
- Saving the word embedding: saving the result in a specific form.

(CBOW Model) The CBOW model contains three layers, including the input layer, projection layer and output layer. The window size used in this paper is 5. The vectors that correspond to each word are first found to be summarized from the input layer to the projection layer. After all of the word vectors in the window are gathered, they are stored in the projection layer, and the mean value is calculated.

(Hierarchical Softmax) Hierarchical Softmax is a key technology that is used in word2vec to improve the performance. In the Huffman tree, the softmax mapping of the hidden layer to the output layer is proceeded step by step along the Hoffman tree and, thus, this softmax is named “Hierarchical Softmax”.

2. Detecting the Flu Period Based on Sentiment Polarity at the Text Level

**Figure 8:**
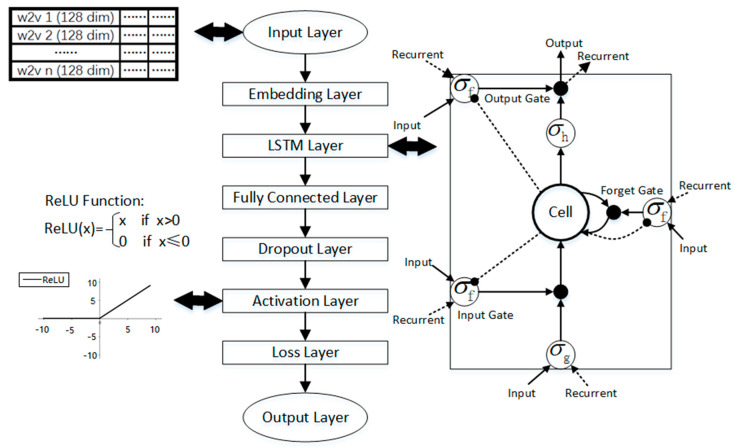
Structure of the Long Short Term Memory Networks Constructed by Layers.

Recurrent Neural Networks (RNNs) perform well in text classification. However, long-term dependence occurs if the interval of two words is overly large. This paper adopts a novel type of RNN called Long Short-Term Memory that works better than traditional RNNs on tasks that involve long time lags. Its architecture permits LSTM to bridge massive time lags between relevant input events (1000 steps and more) [65]. Figure 8 shows the structure of the network with 8 main layers, and we describe each layer below.

**Figure 9:**
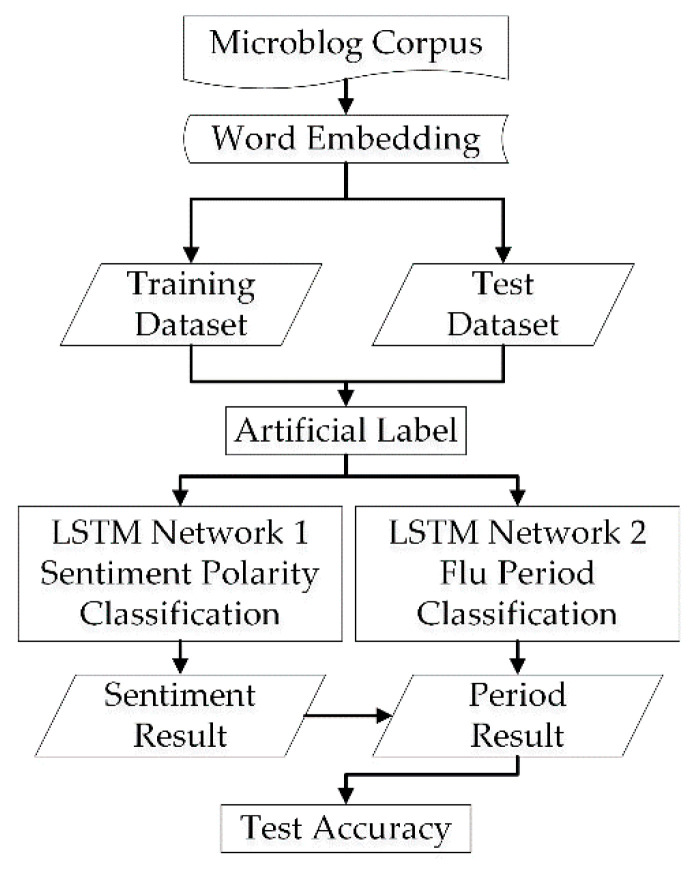
Process of Flu Period and Sentiment Classification.

Two LSTM neural networks in parallel are built to perform binary classification for sentiment classification and flu-period classification in this section. The input is the word embedding trained by the word2vector based on the corpus of flu-related weibos. The entire process is shown in Figure 9.

#### 2.2.3. Construction of LSTM for Sentiment Polarity and Flu Period Classification

The LSTM network is used to construct two neural networks to classify flu-related weibos. The first is to classify the sentiment polarity. The other intends to classify the period of the flu bloggers. Each neural network consists of 8 main layers, as follows.

(Input layer and masking layer) The first layer is the input layer, which uses 128-dimension vectors by means of the word2vector algorithm. The mask value is set to 0 in the masking layer.

(LSTM layer) Each LSTM unit is a storage unit that controls the passage or filtering of information through the three gates, to alter the cell state. “Implementation” is set to 2 to combine the input gate, forget gate, and output gate into a single matrix for more efficient operations.

(Fully connected layer) The core operation of the full connection is the matrix vector product. The essence is a linear transformation from one feature space to another feature space. A dense layer is used.

(Dropout layer) The settings of this layer are mainly to prevent overfitting in neural network training. The parameters of this layer are set to 0.3, which implies that the unit that was transferred from the LSTM layer will be randomly discarded by 30% during training, leaving 70% of the units used.

(Activation layer) The activation function in this layer is “ReLU” (Rectified Linear Units), and as a result, the convergence rate of the model is maintained at a steady state.

(Loss layer and output layer) Since the classification of the sentiment polarity and the period are all binary classifications, binary cross entropy is agreeable as a loss function, with the accuracy rate as a metric of the model. The classification result and test score are received through the output layer.

### 2.3. Data Description

According to the 41st China Statistical Report on Internet Development published by the China Internet Network Information Center (CNNIC), Weibo has 316.01 million users and a user usage rate of 40.9% on social media by December 2017 [66]. Sina Weibo has maintained the top rank in China’s weibo. According to the second quarter earnings released by Sina, monthly active users from Sina Weibo reached 431 million by 30 June 2018, outstripping Twitter as the world’s largest independent social media company in terms of user scale [67]. Sina Weibo has always been the data source of various types of major events and emergencies in China and has a far-reaching scope of dissemination and important social influence. This study uses web-based social media data in Sina Weibo. The details are clearly shown in Table 1.

We collected the texts that contained the keywords, namely, “flu (Gan Mao)”, “influenza (Liu Gan)”, “cough (Ke Sou)”, “fever (Fa Shao)”, “sneeze (Pen Ti)” and “nasal congestion (Bi Sai)” in 2016 and 2017 through a Sina Application Programming Interface (API). We purchased the official data collection service from Gooseeker. Gooseeker is an authorized API of Sina Weibo. The data do not include private information such as personal name, gender, age, etc., and do not endanger privacy and other related issues. All data can be used legally. However, the data include a large amount of advertising and unrelated material. Thus, the Support Vector Machine (SVM) is used to filter out unrelated flu weibos. Ultimately, 100,000 flu-related weibos were chosen randomly as a word2vector training corpus in which 10,000 weibos in 2016 and 10,000 weibos in 2017 were randomly selected as a neural network classification dataset of sentiment polarity and period. In what follows, the 20,000 weibos are labeled according to the following rules. In terms of sentiment polarity, “0” represents positive sentiment, whereas “1”indicates negative sentiment. In terms of the period, “0” indicates the infectious period, while “1” represents the recovered period. The dataset was divided into 4 labeled groups with a total of 12 people involved. Every three members were in one group. Each group was assigned 5000 weibos. Each member in one group was required to label all 5000 weibos without communication to intentionally make the marked category accurate since artificial annotation has a certain subjectivity. The blog was thought to be invalid if the results were inconsistent among the 3 members. After the process of cleaning and arrangement, 15,301 weibos were valid, with the remaining 4699 weibos invalid. We randomly chose 70% of the 15,301 valid weibos as the neural network training set and 30% as the test set.

## 3. Results

### 3.1. Relationship between Sentiment Polarity and Flu-Period State at the Word Level

First, a word2vector corpus was built from the 100,000 flu-related weibos, including 50,000 weibos from 2016 and 50,000 weibos from 2017, by removing irrelevant stop words and symbols. The word frequency of the remaining words was calculated to generate word embedding by similarity and distance between words. Each word consists of a 128-dimensional vector.

One-hundred ninety-five words ranked first and associated with the flu were screened out from the vocabulary and divided into four classes, which represent the two types of flu period (infectious and recovered) and two types of sentiment polarity (positive and negative). The words in the infectious period mainly describe flu confrontation and symptoms. The recovered period describes the status of improvement or remission of the flu. One-hundred ninety-five words in four classes can be found in Table 2.

**Figure 10:**
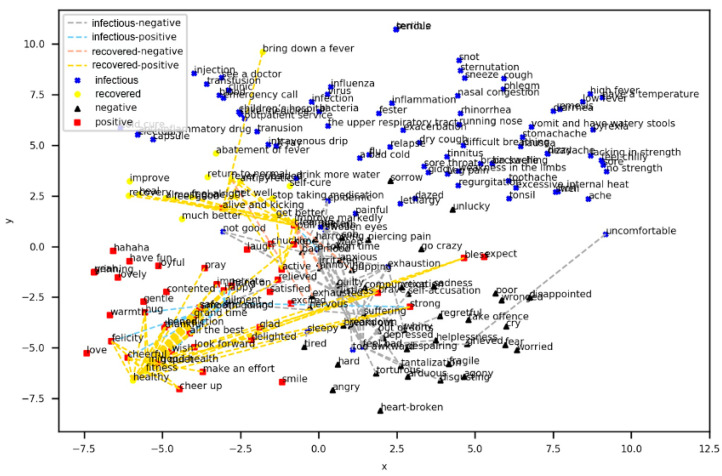
Two-dimensional Word Embedding Scatter Plot.

To display the distribution of the 195 words in two-dimensional space, we used t-distributed Stochastic Neighbor Embedding (t-SNE) to reduce the dimension of the word embedding. The scatter plot was drawn by the two-dimensional coordinates of each word in Figure 10. Each point is a word, with a total of 195 points. It is clear that the words that denote positive sentiment and the recovered period are clustered together, and the words in the negative sentiment and infectious period are clustered together closely. In addition, between these 195 words, this paper calculates the similarity between every two words of the flu period and the sentiment polarity based on word2vector. Additionally, a similarity greater than 0.6 was reserved, which is represented by the connections in Figure 10. Therefore, as seen from Figure 10, there are four types of connections, which are divided between the infectious period and negative sentiment, infectious period and positive sentiment, recovered period and negative sentiment, and recovered period and positive sentiment. This paper also performed statistical analysis on these four types of lines in the scatter plot. When the similarity is greater than 0.6, it is found that there are 53 links between the infectious period and negative sentiment and more than 16 links between the infectious period and positive sentiment. Additionally, there are 81 links between the recovered period and positive sentiment and more than 7 links between the recovered period and negative sentiment. Obviously, the infectious period is much more similar and closer to negative sentiment than to positive sentiment. In contrast, the recovered period is much more similar and closer to positive sentiment than to negative sentiment.

**Figure 11:**
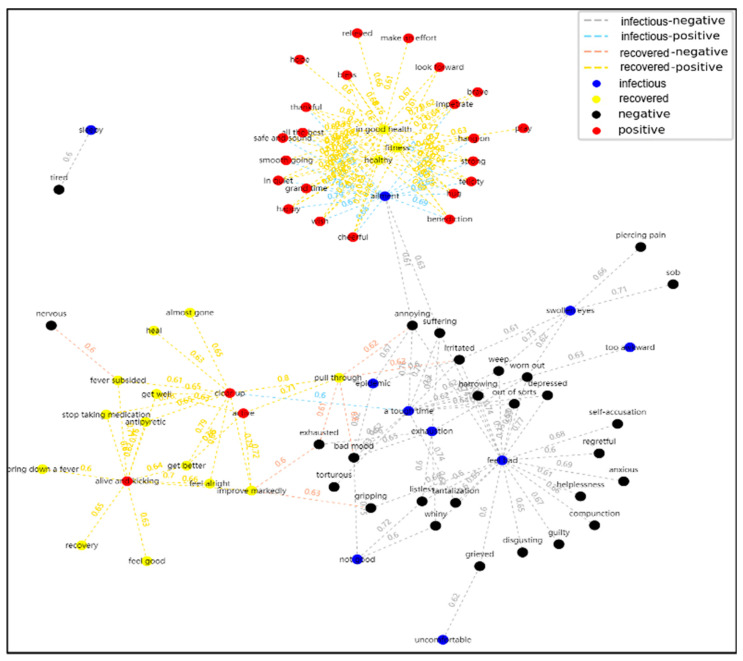
Force-Directed Graph of Words’ Similarity.

To demonstrate the relationship between these 195 words and their similarity, a Force Directed Graph of Words’ Similarity is shown in Figure 11, where a total of 195 nodes represent the 195 words. The nodes are divided into four categories, and the edges between the nodes are also divided into four categories, the same as in the legend in Figure 10. The force of the nodes is the similarity between the two words. It can be clearly seen from Figure 11 that most of the edges around recovered nodes, such as health and fitness, are all positive nodes, such as cheerful and happy. Additionally, positive nodes, such as active, alive and kicking, are connected with recovered nodes, such as get well and heal. In addition, as can be seen from the similarity forces, these three recovered nodes—in good health, healthy, and fitness—which indicate a healthy status, are connected to more positives nodes than these words are, which indicates that the blogger is recovering but not yet healthy. This finding shows that the healthier the bloggers are, the more positive sentiments they have. Additionally, infectious nodes, such as feel bad and uncomfortable, are mutually connected to negative nodes, such as bad mood and anxious. Among the infectious nodes, from uncomfortable to not good to feel bad, the more serious the disease is, the more the negative nodes are connected.

**Figure 12:**
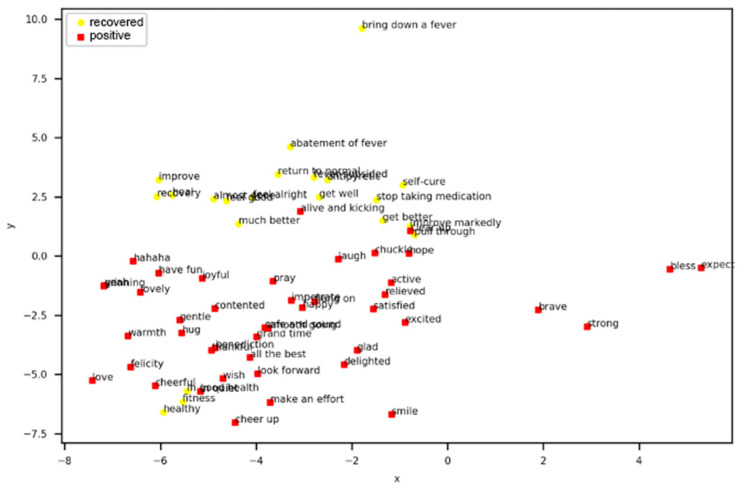
Words of Positive Sentiment and Recovered Period.

**Figure 13:**
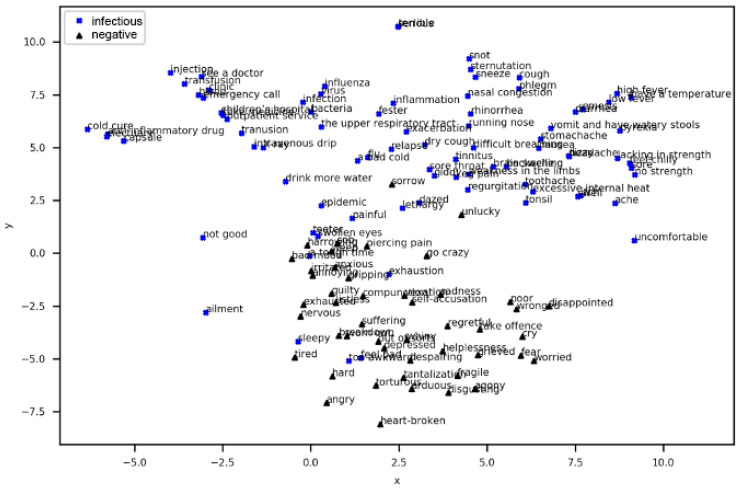
Words of Negative Sentiment and Infectious Period.

To locate the word of each point clearly, the scatter of the points for the positive sentiment and recovered period is plotted in Figure 12. The scatter of the points for the negative sentiment and infectious period is shown in Figure 13. It can be seen from Figure 12 and Figure 13 that semantically related words and words with similar meanings are close to each other. Since the number of words that describe the recovered period is significantly lower than the number that describe the infectious period and the meanings of the words that describe the recovered period are mostly similar, it can be determined that the words of the recovered period and positive sentiment are clustered in the second and third quadrants, as shown in Figure 12. The distribution of the words that represent the infectious period and negative sentiment appear to be more scattered in Figure 13, which denotes that these two classes are more relevant.

**Figure 14:**
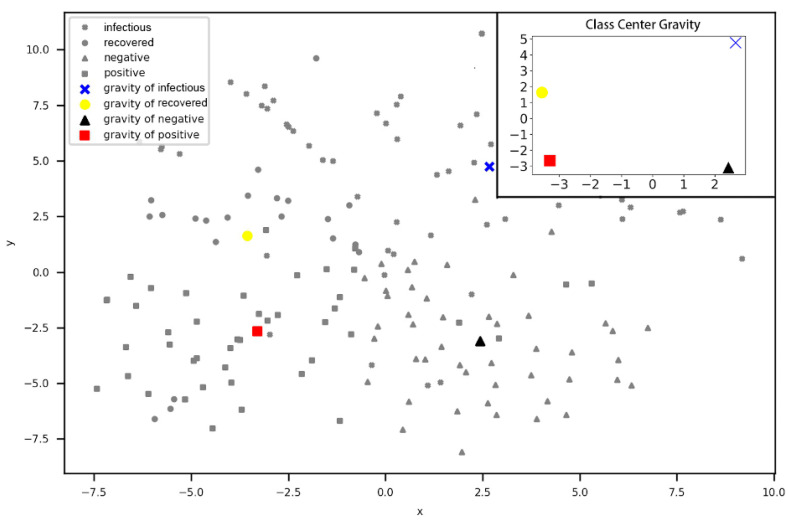
Class Center Gravity Scatter.

To measure the interclass distance of these four categories in the scatter, this paper adopts two sample class distances to compare the relationship between the sentiment polarity and the flu period. The first distance is a centroid cluster, which measures the interclass distance by the distance between the two variables’ mean value. The coordinates of each class’s center gravity are presented in Table 3. The scatter of the class center gravity is plotted through the two-dimensional coordinates of the four categories shown in Figure 14. Figure 14 clearly determines the distribution of four types of class center gravity.

Afterwards, we calculated the Euclidean distance matrix of the center gravity in the following section. From the matrix, the distance between the recovered period and positive sentiment is 4.297, while the distance between the recovered period and negative sentiment is 7.632, which significantly shows that the recovered period is closer to positive sentiment. In terms of the infectious period, the distance to the positive sentiment is 9.521, and the distance to the negative sentiment is 7.867, which indicates that the infectious period is closer to the negative sentiment.

$$\begin{array}{c} \mathrm{Recovered}\\ \mathrm{Infectious}\\ \mathrm{Positive}\\ \mathrm{Negative} \end{array}\left[\begin{array}{cccc} 0 & 6.960 & 4.297 & 7.632\\ 6.960 & 0 & 9.521 & 7.867\\ 4.297 & 9.521 & 0 & 5.753\\ 7.632 & 7.867 & 5.753 & 0 \end{array}\right]$$

In addition, another interclass distance measurement method was selected to further verify the correctness of the conclusion, which is known as the between-group linkage and which measures the interclass distance by the average distance between the two categories of individuals. The Euclidean distance matrix is shown in the following section.

$$\begin{array}{c} \mathrm{Recovered}\\ \mathrm{Infectious}\\ \mathrm{Positive}\\ \mathrm{Negative} \end{array}\left[\begin{array}{cccc} 0 & 8.739 & 6.364 & 8.724\\ 8.739 & 0 & 10.806 & 9.376\\ 6.364 & 10.806 & 0 & 6.977\\ 8.724 & 9.376 & 6.977 & 0 \end{array}\right]$$

This finding implies that the conclusion is consistent with the above. The recovered period is closer to the positive sentiment, and the infectious period is closer to the negative sentiment.

### 3.2. Classification of Flu Period Based on Sentiment Polarity at the Text Level

**Figure 15:**
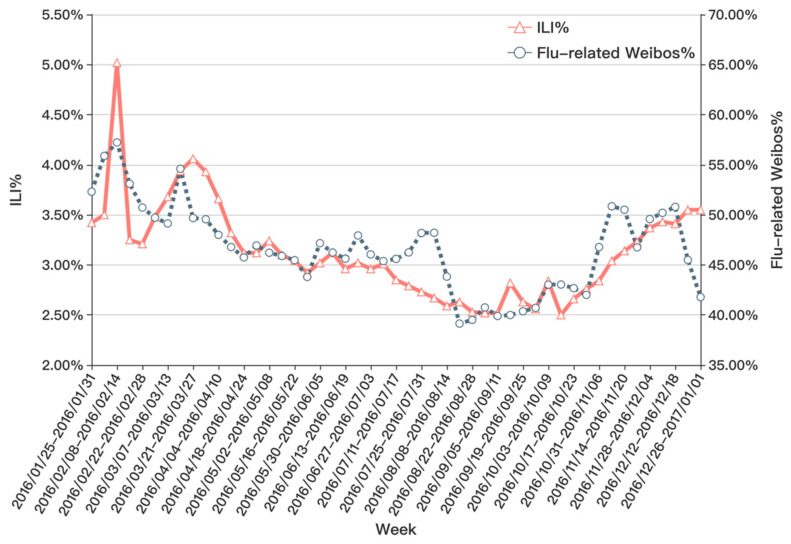
Trend Comparison of Flu-related Weibos% and influenza-like illness (ILI)%.

The flu-related information perceived by social media can detect trend changes and peak points earlier than traditional methods [8,9,19,43]. We also compare the trend of the flu-related weibos ratio and ILI% from the CDC. It can be seen in Figure 15; the flu data perceived on social media can reflect the trend of official ILI data and report changes and peaks earlier in certain weeks.

Two LSTM neural networks were built to classify the sentiment polarity and flu-period in this paper. The input is the word embedding trained by the word2vector based on the corpus of flu-related weibos. It is worthwhile to note that the dimension of the word embedding for the LSTM input is the original word embedding training, which resulted in 128 dimensions. The output of the network is 0 or 1. For the sentiment classification, the output “0” represents negative sentiment, while the output “1” denotes positive sentiment. For the flu-period classification, the output “0” implies that the blogger is infected, while the output “1” implies that the blogger is recovered.

In the LSTM for sentiment classification, the accuracy of the test set reached 0.844 after 30 steps of training. In the LSTM for flu-period classification, the accuracy of the test set reached 0.876 after 30 steps of training. The statistical result of the test set of 4590 weibos was counted to compare the relationship between the flu-period and the sentiment polarity. The prediction result was 876, in which the weibos simultaneously show positive sentiment and the recovered period, shown in Table 3; the other types of prediction results are also shown in Table 3. Apart from these findings, we also counted the number of correct weibos to predict the flu-period in the four types of prediction results. The statistics are shown in Table 4.

The overall accuracy rate is shown as follows,

(7) $${\mathrm{Acc}}_{1}=\frac{\left(831+3190\right)}{\left(1278+3312\right)}=0.876$$

Weibos for both positive sentiment and the recovered period and weibos for both negative sentiment and the infectious period were predicted by the LSTM neural network for a total of 3832 pieces. The accuracy rate is calculated as follows,

(8) $${\mathrm{Acc}}_{2}=\frac{\left(655+2893\right)}{\left(876+2956\right)}=0.926$$

Compared to the two results, it can be determined that the accuracy rate increases to 0.926 when the result of the sentiment classification is added, which indicates that the flu period has a certain correlation with sentiment polarity and the classification accuracy of the flu period improves.

## 4. Discussion

The shortcomings of traditional data are evident since they are manually collected and time-consuming, which leads to high labor costs [8,9]. In addition, traditional methods based on clinical data make it challenging to shed light on the current situation and predict future developing trends [41]. Along with the widespread use of the Internet, social networking data, including web-based epidemiological data, have had explosive growth. Data from social networks show apparent advantages in several respects, such as being real-time and having time-sharing, along with a broad scope of data coverage [31,32,33,34]. In terms of the scale of users, Sina Weibo’s monthly active users reached 431 million on 30 June 2018, overtaking Twitter, which makes Sina Weibo the world’s largest independent social media platform [67]. Sina Weibo is the most popular social media platform in China for the public to share opinions and disseminate information about emergencies and major social events. Therefore, Weibo has a far-reaching scope of dissemination and is an important social influence. Therefore, the use of web-based social media data growth is an imperative trend to use for effective disease control and prevention. Weibo messages carry rich and meaningful implications. Previous approaches in flu state detection through social media have yielded outstanding achievements but have some limitations at the same time. Most obviously, the semantic information was seldom considered; this information might be important for flu detection [50].

However, to our knowledge, this area of study has serious limitations. For example, bloggers experience different flu periods of the latent, infectious, or recovered kind, and their sentiments correspond to the different periods. In reality, most bloggers who are ill are usually negative, while bloggers in recovered periods are active and optimistic. Therefore, omitting the key flu period and non-differentiating Weibo messages data could lead to data contamination and misleading conclusions. It is important to investigate the relationship between the flu period and the sentiment polarity, to make it possible to be conducive to accuracy for classifying the flu period, which directly results in accurately estimating the number of patients in different flu periods. This approach would also help the CDC to take early action for disease control and prevention.

This paper aims to detect the flu period with sentiment polarity at the word and text level based on Sina Weibo data (web-based social media platform), and it proposes optimization suggestions for optimizing the disease detecting process. Several important findings are produced. (1) Social media is a promising and powerful data platform to detect flu patients by earlier discovery rather than traditional medical data. Their periods can be further sorted into infectious and recovered, as mined from social media. (2) The semantic information varies from the weibo texts posted by patients in different flu periods. The interclass distance between the recovered period and positive sentiment is closer than between the recovered period and negative sentiment, and the interclass distance between the infectious and the negative is closer than that of the positive. Additionally, it was noted that the healthier the bloggers are, the more positive sentiments they have. The more serious the flu is, the more that bloggers are connected with negative emotions. (3) A multichannel disease detection model is developed in this study to evaluate and classify the flu period with an accuracy of up to 0.926 based on the LSTM network. Our optimized model effectively improves the classification accuracy of the flu period after adding the sentiment classification results.

The research findings have important theoretical implications. (1) The previous literature investigates the sentiment and disease predictions separately. This paper examines the relationship between sentiment and disease detection. We found that by adding sentiment factors, the classification accuracy is improved remarkably, from 0.876 to 0.926. (2) This paper explores the relationship between the sentiment polarity and the flu-period at two levels of words and text, combining the methods of word2vector and LSTM, which have been used rarely for disease surveillance studies. (3) This research proposes a complete theoretical framework based on web-based social media data. The use of this model can be extended to many aspects, such as monitoring chronic and mental diseases.

This study also has important practical implications. (1) This paper optimizes the disease detecting process and establishes multichannel surveillance measures for CDC decision making. (2) This paper will monitor a larger range of infected population. Furthermore, it can identify patients in advance who are not aware of disease. (3) The previous weibo text processing classifies only flu-related weibos and unrelated weibos. This paper further divides flu-related weibos into two periods: recovered and infectious. Research outcomes improve the reliability and accuracy for the prediction of flu trends. Both point (2) and point (3) not only help the CDC to detect disease information in real time but also provide a novel method for disease information management. (4) The conclusion supports the expansion of the number of neural network training sets, eliminating some of the high cost of manual labeling. The classification results of the flu-period can be replaced in the model to increase the amount of training set data, which enables the LSTM neural network to fully learn to better characterize the model.

## 5. Conclusions

Timely and reliable flu monitoring is an important basis for successful control of the spread of disease and mitigation of the associated damage. However, due to its high contagiousness and rapid spread, the flu epidemic has caused great difficulties in prevention and surveillance. With the rapid development and popularity of web-based social media platform data, Sina Weibo, one of the world’s largest social media companies, has become an ideal data source to make real-time, low-cost surveillance possible as an early warning of outbreaks and an adjunct to traditional methods of investigation. According to the latest estimate by the United States Centers for Disease Control and Prevention (US-CDC), as many as 650,000 people worldwide die from seasonal flu-related respiratory diseases each year. It is evident that the flu imposes a heavy burden on the international community, and the flu’s global, social and economic costs are considerable. It is worth noting that improving the ability to monitor infectious disease is the key to further strengthening management capacity of the health system and organizing a massive flu outbreak response.

However, most traditional epidemiological surveillance methods adopt clinical data through manual information collection, showing the shortcomings of high labor costs but also causing a lag in data timeliness. The data limitation makes it difficult to understand the current situation, which is critical for flu trend forecasting. Social media data increase at a rapid pace, including epidemiological data, which offer benefits in terms of timeliness and magnitude. Sina’s 2018 quarter earnings report that the number of monthly active users of Sina Weibo is larger than that of Twitter, which makes it the world’s largest independent social media company in terms of user scale. Sina Weibo has always been the starting data source of various types of major emergencies in China, and its commercial value has been continuously promoted and has great potential.

This paper explores the relationship between the flu-period and sentiment polarity from two levels based on Sina Weibo data. To be specific, at the word level, we used word2vector to create the flu-related weibos corpus and the t-SNE method to reduce the dimension. The centroid cluster and between-group linkage were jointly used to measure the distance between the four classes, thus visually showing the relationship between the sentiment polarity and flu-period. At the text level, the sentiment polarity and flu-period of flu-related weibos were classified by the LSTM networks, respectively. We counted the classification results as both belonging to the infectious and negative sentiment as well as to the recovered and positive sentiment, and we calculated the accuracy rate. We then compared the rate with the overall flu-period classification accuracy to observe the differences. This paper proposes an integrated conceptual framework and practical methods for optimizing the disease detection process with fast information, early discovery, added infected cases and high accuracy. These contributions are described in detail as follows:

First, in theory, this paper integrates various channels for detecting infectious diseases in real time with fast information. In addition to the clinical data and search engine data, the detecting data obtained through social media can also provide prompt and time-sharing disease information to the Centers for Disease Control and Prevention (CDC). The monitoring mechanisms operate in real time, which can help the CDC fully prepare for the next round of prevention and control.

Second, in practice, social media enables the early discovery of disease infection. The sooner the disease is diagnosed, the easier it is to properly treat and controlled. The CDC is committed to pursuing early detection of diseases. Through social media platforms, we can detect the spread and severity of a disease earlier than search engines and the CDC. When diseases break out, the patient might not be aware of them but could post on Twitter or Weibo. The behavior would be recorded by social media sensors. Based on human behavioral theory, the data possess unique value for detecting disease trends.

Third, social media is adept at tracking more patients than traditional clinic data. Larger infectious populations can be monitored by social media than with clinic data. Influenza-like illnesses (ILI) published by the CDC are measured according to outpatient statistics when fevers are higher than 38 degrees and are accompanied by a cough or sore throat. However, a considerable number of people often choose not to go to the hospital for treatment when they have the flu or might buy medicine from a pharmacy by themselves, which cannot be counted in ILI measurements. Social media can detect these patients, which could result in a larger amount of meaningful data being collected, and thus, these data could lead to more reliable prediction of disease outbreaks.

Fourth, this paper detects disease periods with observably high accuracy, which could directly result in significant differences in treatment and disease control measures. Targeting the disease period precisely helps clinical managers to improve the treatment effect and reduces the prevention cost by rationally allocating resources, such as medical personnel and medicine as well. This paper can not only detect whether the patient has the flu but also classify the flu period, infectious or recovered period, which lays the foundation for predicting future flu trends. It also provides another data source to assist the CDC in managing disease information.

Fifth, in terms of theoretical contributions, this paper investigates the relationship between sentiment polarity and the flu period at different word and text levels by combining the word2vector and LSTM methods, thereby carrying out interdisciplinary research in the fields of sentiment analytics and health informatics. In addition, this paper provides an effective solution for artificially labeling a training set. High-accuracy weibo texts can be used to boost the size of the training set, thus saving time and labor costs.

In future work, we need to study a wider range of data since the current data only cover two years, 2016 and 2017. Moreover, this paper compares the trend of official ILI data from the CDC and flu-related data from social media in 2016. We will examine more valuable disease information from social media-based data on a larger scale.

## Figures and Tables

**Table 1:** Data Description.

Category	Field Name	
Tweet’s information	URL, released time, title, text	
Blogger’s Information	blogger’s ID, nickname of the blogger	
Resource	Sina Weibo	
Keywords	flu (Gan Mao), influenza (Liu Gan), cough (Ke Sou), fever (Fa Shao), sneeze (Pen Ti), nasal congestion (Bi Sai)	
Amount of word2vector training corpora	In 2016	50,000
	In 2017	50,000
Amount of labeling sets	In 2016	10,000
	In 2017	10,000
Total valid amount	15,301	
LSTM training set	10,711	
LSTM test set	4590	

**Table 2:** Flu-related Words.

Label	Words
infectious	uncomfortable, not good, ailment, no strength, ache, too awkward, feel bad, serious, sleepy, exhaustion, a tough time, high fever, low fever, pyrexia, diarrhea, emesis, vomit and have watery stools, sneeze, phlegm, dry cough, nasal congestion, difficulty breathing, sore throat, running nose, a bad cold, excessive internal heat, relapse, swell, sore, itch, clinic, children’s hospital, emergency call, see a doctor, transfusion, blood, outpatient service, take medicine, injection, drink more water, transfusion, headache, dizzy, backache, weakness in the limbs, stomach ache, leg pain, giddy, terrible, exacerbation, brain swelling, nausea, regurgitation, tonsil, anti-inflammatory drug, capsule, electuary, painful, fester, tinnitus, toothache, sternutation, cough, lacking in strength, intravenous drip, bacteria, influenza, infection, epidemic, the upper respiratory tract, feel chilly, swollen eyes, X-ray, dazed, lethargy, teeter, have a temperature, flu, rhinorrhea, snot, cold cure, inflammation, virus.
recovered	bring down a fever, much better, improve, healthy, recovery, almost gone, feel good, heal, feel all right, get better, fever subsided, antipyretic, abatement of fever, return to normal, stop taking medication, in good health, fitness, get well, improve markedly, pull through, self-cure.
negative	sadness, cry, go crazy, poor, disappointed, tired, heart-broken, agony, worried, unlucky, wronged, grieved, breakdown, disgusting, angry, torturous, sorrow, hard, arduous, piercing pain, sob, anxious, self-accusation, vexation, compunction, fear, gripping, exhausted, weep, worn out, fragile, suffering, helplessness, tantalization, nervous, take offence, guilty, regretful, despairing, whiny, harrowing, depressed, annoying, out of sorts, irritated, listless, bad mood.
positive	quiet, happy, thankful, wish, clear up, alive and kicking, hope, expect, laugh, love, smile, delighted, cheer up, ha ha ha, make an effort, lovely, grinning, felicity, warmth, cheerful, strong, glad, excited, pray, bless, impetrate, look forward, chuckle, satisfied, joyful, active, all the best, smooth going, hang on, have fun, yeah, contented, hug, gentle, safe and sound, benediction, grand time, brave, relieved.

**Table 3:** Class Center Gravity Coordinates.

Label	X	Y
Infectious	2.665	4.758
Negative	2.434	−3.105
Positive	−3.302	−2.661
Recovered	−3.553	1.629

**Table 4:** LSTM Test Set Result.

Period and Sentiment		Positive	Negative	Total
Recovered	total	876	402	1278
	correct	655	176	831
Infectious	total	356	2956	3312
	correct	297	2893	3190
